# What clinical and laboratory parameters determine significant intra abdominal pathology for patients assessed in hospital with acute abdominal pain?

**DOI:** 10.1186/1749-7922-2-26

**Published:** 2007-09-25

**Authors:** Saleh M Abbas, Troy Smithers, Etienne Truter

**Affiliations:** 1 Middlemore Hospital, Department of surgery, Auckland, New Zealand; 2 Roturoa Hospital, Department of surgery, Rotorua, Hospital Road, New Zealand

## Abstract

**Background:**

Abdominal pain is a common cause for emergency admission. While some patients have serious abdominal pathology, a significant group of those patients have no specific cause for the pain. This study was conducted to identify those who have non-specific abdominal pain who can be either admitted short term for observation or reassured and discharged for outpatient management.

**Patients and methods:**

A prospective documentation of clinical and laboratory data was obtained on a consecutive cohort of 286 patients who were admitted to a surgical unit over a nine month period with symptoms of abdominal pain regarded severe enough for full assessment in the casualty department and admission to a surgical ward. The patients were followed until a definite diagnosis was made or the patient's condition and abdominal pain improved and the patient discharged. The hospital where the study took place is a small peripheral general hospital draining a population of 120,000 people in a rural area in New Zealand.

**Results:**

There were 286 admissions to the emergency department. Logistic regression multivariate statistical analysis showed that guarding raised white cells count, tachycardia and vomiting were the only variables associated with significant pathology.

**Conclusion:**

Patients with no vomiting, no guarding, who have normal pulse rates and normal white cell counts are unlikely to have significant pathology requiring further active intervention either medical or surgical.

## Introduction

Abdominal pain is very common presentation to emergency department. It is vital that the physician has an understanding and be familiar with the presentations of common diseases that cause abdominal pain [1,2]. Patients with acute abdominal pain are a heterogeneous group that consumes a great deal of a surgical department's resources. To streamline efficiency and provide maximum cost effectiveness it would be of benefit to identify clinical and laboratory parameters in patients admitted with acute abdominal pain that would indicate no significant intra abdominal pathology and thereby encourage early discharge back to the community [3,4].

Women of childbearing age commonly present with right iliac fossa pain, most of them do not have appendicitis [4]. Those who do not have the classical features of appendicitis with no evidence of peritonism on examination can be safely managed by active observation or diagnostic laparoscopy [4,5]. This study was designed to identify the clinical and laboratory red flags. Recognizing these red flags in the history and physical examination and the initial imaging and laboratory findings helps to determine which patients may have a serious underlying disease process, and therefore warrant more expedited evaluation and treatment.

## Patients and methods

Patients assessed at the emergency department at Rotorua Public Hospital with acute abdominal pain requiring admission to the surgical unit were enrolled in the prospective study.

Patients were examined by the admitting surgical team after taking a thorough history, Relevant points in the history included the patient's gender, duration of pain, site of pain, character of pain, fever, loss of appetite, change in bowel habit, vomiting, abdominal distension and urinary or genital symptoms.

Factors in the clinical examination that were considered of significant contribution to the final diagnosis included temperature, tachycardia, and abdominal tenderness and localized guarding.

Data was collected prospectively on an electronic database. Patients were followed up with further investigations that included ultrasound scan, CT scan and diagnostic laparotomy as was clinically indicated to ascertain the final diagnosis. All patients were admitted for observation or surgery if indicated.

Non-specific abdominal pain was defined as an abdominal pain in right iliac or hypogastric area lasting more than 6 hours and less than 8 days, without fever, leukocytosis, or obvious peritoneal signs and uncertain diagnosis after physical examination and baseline investigations including abdominal sonography; provide they have totally settled or underwent diagnostic laparoscopy that proved to be normal.

### Analysis

Analysis was performed to evaluate the relative contribution and specificity of each individual factor to a definitive final diagnosis that led to identification those who had no definitive diagnosis. The data analyzed using SPSS^® ^version 10.5. Univariate analysis was performed using simple linear regression; multivariate analysis was performed using logistic regression analysis.

## Results

There were 286 patients over three months period of the study, 201(70%) females). Median age is 37 years. The causes of abdominal pain are shown in (table 1). Non-specific abdominal pain was the diagnosis in 98 patients (34% of total). 72 (73%) of them were females; the non-specific nature of the pain was made by clinical resolution of the symptoms or diagnostic laparoscopy. None of those patients with nonspecific pain had guarding or vomiting, and only five of them had raised white cells count. Non-surgical cause was seen in 47 patients (table 2).

**Table 1:** Surgical causes of pain

Diagnosis	Number of patients
Acute appendicitis	36
Biliary colic	9
Cholecystitis (normal liver function test)	12
Diverticulitis	14
Probable Sub-acute small bowel obstruction (minimal X rays findings)	7
Colon cancer	4
Peptic ulcer disease	6
Pancreatitis with normal amylase	3

**Table 2:** Non surgical causes of pain.

Diagnosis	Number	Method of diagnosis
Acute pelvic inflammatory disease	8	Diagnostic laparoscopy
Rupture ovarian follicle	5	Diagnostic laparoscopy
Renal colic	12	CT scan
Urinary tract infection	8	Midstream urine examination and culture
Gastroenteritis	8	Clinical evidence (diarrhea and vomiting)
Clostridium Difficile colitis	1	Faecal culture
Mesenteric lymphadenitis	5	Open appendicectomy

Univariate analysis showed important factors that predict acute surgical pathology are guarding (p = <0.0001), raised WBC (p = <0.0001), tachycardia (pulse rate greater than 100 per minute) (p < 0.0001), temperature (p < 0.0001), rigors (p = 0.0008), lack of appetite (p < 0.0001) and vomiting (p < 0.0001). Table 3

**Table 3:** Univariate analysis

Sign	P Value
Character of the pain	0.101
Tenderness	0.042
Guarding	<0.0001
Leucocytosis	<0.0001
Tachycardia	<0.0001
Raised temperature	<0.0001
Rigors	0.0008
Lack of appetite	<0.0001
Vomiting	<0.0001
Patient in distress	0.6135
Gender	0.223
Altered bowel habit	0.25
Location of the pain	0.9999

Multivariate analysis was conducted using logistic regression; it showed he only significant predicting factors for acute surgical diagnosis are Heart rate (p = 0.048), guarding (p = 0.0009), WBC (p = 0.016) and vomiting (p = 0.008). Table 4.

**Table 4:** Multivariate analysis

Sign	P value
Gender	0.882
Character of the pain	0.070
Duration	0.915
***Vomiting***	0.008
Altered bowel habit	0.940
Lack of appetite	0.579
Urinary symptoms	0.163
Raised temperature	0.917
Rigors	0.345
***Tachycardia***	0.048
Abdomen tender	0.112
***Guarding***	0.0009
***Leucocytosis***	0.016

## Discussion

The current study shows that patients who present with abdominal pain with no vomiting, guarding or raised white cell count are unlikely to have significant intra abdominal pathology and therefore can be considered for early discharge back to the community. Abnormal liver function test was helpful to point out the possibility of biliary colic, so was microscopic hematuria that may reflect urinary tract pathology.

Complaints of acute abdominal pain are common emergency department presentations. Many of these conditions prove to be benign and self-limiting illness, which has no clear explanation. Tenderness and peritonism in the right iliac fossa are not specific for appendicitis but may help to narrow the differential diagnosis in patients with right iliac fossa pain [6,7]. However high WBC counts and left shift are strongly associated with appendicitis in children aged 1 to 19 years indeed for children older than 4 years with lower abdominal pain; the most common diagnosis in the setting of an elevated WBC count is acute appendicitis [8].

In females of childbearing age, the presence or absence of bilateral tenderness pain migration and vomiting may help to differentiate acute appendicitis from acute pelvic inflammatory disease [9] where patients tend to have raised white cells count and demonstrate signs of peritonism, which can make the differentiation between the two diagnoses on clinical grounds difficult. Mesenteric adenitis is very difficult to diagnose clinically and commonly patients undergo treatment for presumed appendicitis [10-12].

Imaging such as ultrasound [13] is helpful for detection of the cause of lower abdominal pain; in clinical centers where CT scan is routinely performed it reduces the rate of both negative appendicectomy and perforated appendicitis, whoever CT scanning has significant radiation dose for children and young adults [9,14] and its not always practical or available. On the other hand elderly patients can have atypical presentations; vascular events are more common in this population, and a wide differential diagnosis needs to be considered [15].

Early diagnostic laparoscopy and treatment results in accurate, prompt, and efficient management of acute abdominal pain. This technique reduces the rate of unnecessary laparotomy and right iliac fossa gridiron incisions and increases the diagnostic accuracy in these patients; however this approach is expensive with significant use of the resources and potential morbidity [5]. Diagnostic laparoscopy also avoids extensive preoperative investigations, averts delays in operative management [16]. Morino et al assessed the role of diagnostic laparoscopy and concluded that laparoscopy compared with clinical observation, early laparoscopy did not show a clear benefit in women with non-specific abdominal pain. They reported higher number of diagnosis and a shorter hospital stay in the laparoscopy group [17,18]. In another study patients who have no evidence of guarding, normal temperature, and normal white cells count were observed; a practice that is proven to reduce the rate of negative appendicectomy [12]. Non-specific abdominal pain is the most common diagnosis in patients admitted with acute abdominal pain (25–35% of all patients), and of those patients only a quarter needs surgical intervention [2,19]. Clinical scores that were developed to recognize acute appendicitis [20] are useful but those score could only be used with clinical judgment, if there is any suspicion of serious pathology the patient should undergo a diagnostic laparoscopy.

The current study shows the likely groups of patients with non-specific abdominal pain are those with normal white cell count, no history of vomiting and absent signs of peritoneal irritation with normal heart rate. Patients who fulfill those findings are suitable for early discharge, which may help to reduce the health care cost for those patients. All patients admitted to emergency department with abdominal pain should be referred for surgical opinion and the diagnosis of nonspecific abdominal pain should only be made after thorough assessment, definite pathology excluded and the patient does not return with the same complaint.

## Competing interests

The author(s) declare that they have no competing interests.
